# Role of aldosterone on lung structural remodelling and right ventricular function in congestive heart failure

**DOI:** 10.1186/1471-2261-11-72

**Published:** 2011-12-02

**Authors:** Andreanne Chabot, Bao Hua Jiang, Yanfen Shi, Jean-Claude Tardif, Jocelyn Dupuis

**Affiliations:** 1 Research Center, Montreal Heart Institute/Université de Montréal, 5000 Bélanger Street, Montreal, Quebec, H1T 1C8, Canada; 2 Department of Medicine, Université de Montréal, 2910 boul. Edouard-Montpetit, Montreal, Quebec, H3T 1J7, Canada

**Keywords:** spironolactone, pulmonary heart disease, pulmonary hypertension, myofibroblasts

## Abstract

**Background:**

The mechanisms of benefit of mineralocorticoid receptors antagonists in congestive heart failure (CHF) are still debated. We hypothesized that aldosterone contributes to pulmonary remodelling and right ventricular (RV) dysfunction associated with CHF by stimulation of lung myofibroblasts (MYFs) proliferation.

**Methods:**

Rats with moderate to large myocardial infarcts (MI) and CHF were studied. Two weeks after MI, spironolactone 100 mg/kg/day (n = 21) or no treatment (n = 24) were given for 3 weeks and compared to sham (n = 8).

**Results:**

Infarct size was similar by ultrasound and pathologic measures in both MI groups.

The MI-untreated group developed important lung remodelling with nearly doubling of dry lung weight (p < 0.01), reduced left ventricular (LV) fractional shortening (16 ± 2% vs. 53 ± 1%; mean ± SEM, p < 0.0001), pulmonary hypertension (RV systolic pressure: 40 ± 3 mmHg vs. 27 ± 1 mmHg, p < 0.01) and RV hypertrophy (RV/(LV + septum): 38 ± 3% vs. 24 ± 1%, p < 0.05). Spironolactone had no effect on these parameters and did not improve LV or RV performance (tricuspid annular plane systolic excursion and RV myocardial performance index) measured by echocardiography. CHF induced a restrictive respiratory syndrome with histological lung fibrosis: this was also unaffected by spironolactone. Finally, isolated lung MYFs did not proliferate after exposure to aldosterone.

**Conclusion:**

Aldosterone does not significantly contribute to pulmonary remodelling and RV dysfunction associated with CHF. Other mechanisms are responsible for the beneficial effects of spironolactone in CHF.

## Background

Pulmonary hypertension (PH) is a frequent complication of congestive heart failure (CHF). Conversely, left heart disease represents the single most prevalent causative factor for PH. PH, especially when associated with right ventricular (RV) dysfunction, reduces exercise capacity and represents an important independent prognostic factor in CHF [1]. At the core of pulmonary adaptations to CHF lies pulmonary structural remodelling with abundant proliferation of alveolar wall myofibroblasts (MYFs) [2-5]. MYFs are star-shaped cells that have a physiological role in growth, development and repair of tissue, and express morphological characteristics of both fibroblasts and smooth muscle cells [3]. The proliferation of MYFs is also associated with collagen and reticulin deposition in the alveolar septa, leading to thickening of capillary endothelial and alveolar epithelial cell basement membranes, which results in reduced lung compliance [3-7]. Although these adaptive mechanisms provide protection against alveolar oedema formation, they induce a maladaptive restrictive lung syndrome, affecting lung mechanics and gas exchange and contributing to the loss of functional capacity of CHF subjects [5,6]. The reduction of total lung capacity is proportional to the severity of heart disease as measured from cardiopulmonary exercise testing [8]. Unfortunately, the biologic determinants of lung structural remodelling associated with CHF are currently unknown.

The aldosterone antagonists eplerenone and spironolactone are currently approved for the therapy of CHF [9-11] and constitute a class I recommendation (useful and recommended) according to the guidelines of the American College of Cardiology/American Heart Association (ACC/AHA) [12]. Despite proven benefits on reducing morbidity and mortality, the exact mechanisms of action of aldosterone inhibition remains debated and several investigators have reported on the underuse of these agents [13]. One of the potential beneficial extra-renal effects of aldosterone inhibition is the reduction of cardiac remodelling [14,15]. Furthermore, the use of spironolactone in CHF has shown positive effects on lung tissues by improving gas diffusion and exercise capacity [16], suggesting that spironolactone could also have some beneficial effect on lung remodelling associated with CHF. Pre-clinical studies have also revealed that aldosterone could play a role in the development of lung fibrosis [17].

Against this background, we hypothesized that aldosterone contributes to lung structural remodelling and the ensuing pulmonary hypertension and RV dysfunction associated with CHF by promoting lung MYFs proliferation and fibrosis. As a corollary, a mechanism of benefit of spironolactone therapy would be to improve lung remodelling, secondary PH and RV dysfunction.

## Methods

The animal ethics committee of the Montreal Heart Institute approved the study protocol. It was implemented following the guidelines of the Canadian Council on Animal research in accordance with the *Guide for the Care and Use of Laboratory Animals *[18].

### Experimental protocol

Myocardial infarction (MI) or sham surgery was performed on male Wistar rats as previously described [2-4,7,19-21]. Briefly, rats were anaesthetized, intubated and put on a rodent ventilator (Harvard Apparatus). Then, a left-sided thoracotomy was realized in order to expose the heart and a ligation of the proximal left anterior descending coronary artery was performed. The sham group underwent the same procedure with the exception of the ligation of the artery.

This study was designed to evaluate animals with CHF and lung remodelling using a previously validated approach based on troponin T measurement 24 hours after coronary ligation [7,19]. Plasma troponin-T ≥ 5.1 μg/L predicts moderate to large infarct size (≥ 30% left ventricular (LV) wall motion abnormality) with a sensitivity of 91% and a specificity of 84% [7,19]. Using this approach we previously demonstrated that these animals develop lung remodelling, PH and RV dysfunction. Blood samples were collected via the subclavian vein, centrifuged and then analyzed by standard electrochemiluminescence immunoassay using the Cobas e 601 (Roche). Two weeks after surgery, the selected rats were randomly divided into 2 groups: MI+spironolactone (Novopharm, 100 mg/kg/day in food; n = 21) and MI-untreated (n = 24). The sham group (n = 8) did not receive treatment. These therapies lasted for 3 weeks and the animals were studies 5 weeks after MI.

### Transthoracic echocardiographic study

Each rat was anaesthetized with 2% of isoflurane to determine LV wall motion score abnormality and LV and RV functions and geometries using a phased-array probe 10S (4.5-11.5 Megahertz) linked to a Vivid 7 system (GE Healthcare Ultrasound) [2].

### In vivo respiratory function tests and hemodynamic measurements

Ketamine (50 mg/kg) and xylazine (10 mg/kg) were administered and the trachea was isolated. A tracheotomy was performed and an endotracheal tube was installed. To determine lung compliance, lung elastance and measure respiratory function, rats were connected to a computer-controlled small-animal ventilator (FlexiVent, Scireq) as previously described [2]. Respiratory pressure-volume (P-V) loops were produced for each animal and analyzed using the Salazar-Knowles equation: **V = A - B·e^-KP^**. Where, **A **is the estimate of the inspiratory capacity, **B **is the estimate of total lung capacity (*P *= 0), **K **is the curvature parameter of P V loop, **P **is the pressure and **V **is the volume.

Using a Powerlab polygraph system (AD Instruments), LV and RV hemodynamic parameters were measured by the isolation and incision of the right jugular vein and carotid artery followed by the insertion of high fidelity pressure catheters (Millar Instruments) in the RV and LV.

### Lung and heart morphometric and histological measurements

Morphometric measurements were performed as previously described [2]. First, the right middle lung was isolated and weighed (wet weight). The lobe was dried for two weeks before measuring dry weight. Pulmonary oedema was determined by the ratio of dry/wet lung weights. The left lung was perfused and fixed with optimal cutting temperature compound (OCT, Sakura), then snap-frozen in liquid nitrogen to finally be frozen in 2-methylbutane for later study.

The hearts were isolated and dissected to separate the left and right ventricles in order to assess RV hypertrophy (RVH) by weight. LV scars were dissected and weighed and their surface area determined by planimetry.

Histological slides of lung and LV tissue from sham (n = 8), MI-untreated (n = 10) and MI+spironolactone (n = 10) were stained with Masson's trichrome. For each slide, 10 random visual fields (10X) were analyzed with the Image-pro Plus 6.2 software (Media Cybernetics) to determine the collagen-staining ratio.

### RT-PCR for osteopontin (OPN) gene expression

Lung samples form sham (n = 8), MI-untreated (n = 10) and MI+spironolactone (n = 10) were prepared and studies. Real-time PCR was performed with the Platinum SYBR Green qPCR Kit SuperMix-UDG (Invitrogen) with use of the MX3005P device (Stratagene). To evaluate the expression of OPN gene in the lungs, the oligos of rat OPN forward [TCAGATGCTGTAGCCACTTG]; rat OPN reverse [TTCACAGAATCCTCGCTCTC]; rat cyclo A housekeeping gene forward [CTGATGGCGAGCCCTTGG] and rat cyclo A reverse [GCCACCAGTGCCATTATG] were used.

### Lung MYFs isolation and proliferation

Lung MYFs of male Wistar rats (175-200 g) were isolated as previously described [2,4]. Briefly, rats were anaesthetized with intramuscular injection of a ketamine/xylazine solution (1 ml/kg). After thoracotomy, the peripheral regions of each lung lobe were dissected and cut into pieces of about 3-5 mm and washed twice in a Hank's Balanced Salt Solution (HBSS) containing 0.5% Ciprofloxacin. Subsequently, tissues were placed in a 0.1% trypsin solution overnight at 4°C with stirring to be digested. The next day, lung tissues were digested some more by up and down pipetting motion in a 0.1% collagenase II solution until solution appeared cloudy, then it was centrifuged at 3000 RPM for 3 minutes at 4°C. The pellets were re-suspended in HBSS and re-centrifuged twice. The final pellet was dissolved in 50 ml of Dulbecco's modified Eagle's medium (DMEM) containing 6.7% Fetal Bovine Serum (FBS), 1.8% Penicillin-Streptomycin, 0.9% Amphotericin B and 0.5% Ciprofloxacin. Lung cells were then plated in T-75 flasks in an incubator (Sanyo Scientific) maintained at 37°C and 5% CO_2_. These are ideal conditions to stimulate cell proliferation. The culture medium (FBS) was changed every 2 days until confluence. MYFs were passaged with Trypsin-EDTA 1X at 37°C and plated in 96-wells plates (1500 cells/200 ul) for CyQUANT cell proliferation assay.

### CyQUANT cell proliferation assay

After reaching confluence again (around 24 hours), the first-pass lung MYFs in 96-wells plate were starved for 24 hours with a serum-free media made of DMEM containing 0.2% ITS (Insulin, Transferrine and Selenium) and 2% Penicillin-Streptomycin. Thereafter, the cells were treated for 48 hours with one of the following three solutions: FBS positive control, ITS negative control and aldosterone (Sigma) 10^-7^M (n = 26) or 10^-6^M (n = 23). After treatment, the culture medium was removed and the 96-wells plates were frozen for at least 72 hours. Cellular proliferation was then determined using the CyQUANT Cell proliferation Assay Kit (Invitrogen) following the manufacturer's instructions.

### Statistical analysis

Parameters from the 3 experimental groups were evaluated by a one-way analysis of variance (ANOVA) followed, when a significant interaction was found (p < 0.05), by the Fisher's post hoc test for multiple groups comparisons. All values were expressed as mean±SEM and values of p < 0.05 were considered to be statistically significant.

## Results

From the moment of randomization to treatment (2 weeks post MI) until the end of study (5 weeks), there was no mortality in any group. Plasma troponin-T concentrations measured 24 h after infarct was similar in the MI-untreated (8.5 ± 0.4 μg/l) and the MI+spironolactone (9.1 ± 0.3 μg/l) groups indicating comparable myocardial injury prior to treatment allocation (Table 1). Five weeks after MI, this translated into moderate to severe CHF as evaluated from hemodynamics and echocardiography.

**Table 1:** LV and RV echocardiographic parameters

	Sham	MI-untreated	MI+spironolactone
LV wall motion abnormality (%)	0	50 ± 5*	50 ± 4*
LV wall motion score index	1 ± 0	1.95 ± 0.06*	1.79 ± 0.07*
LV end-diastolic dimension (mm)	8.2 ± 0.1	11.2 ± 0.2*	10.8 ± 0.2*
LV end-systolic dimension (mm)	3.9 ± 0.1	9.5 ± 0.3*	9.0 ± 0.3*
LV end-diastolic area (mm^2^)	49 ± 1	92 ± 3*	85 ± 3*
LV end-systolic area (mm^2^)	11 ± 1	64 ± 4*	58 ± 4*
TVc-o (msec)	103 ± 10	119 ± 6	121 ± 6
RV end-diastolic dimension (mm)	3.2 ± 0.1	3.4 ± 0.1	3.3 ± 0.2

LV, left ventricular; RV, right ventricular; TVc-o, tricuspid valve closing to opening.

*p < 0.0001 vs. Sham.

### Effects of spironolactone on LV function and LV remodelling

**Figure 1:**
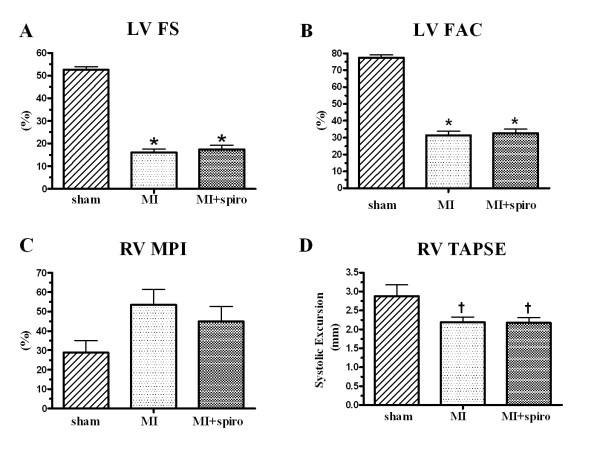
**Effect of spironolactone on echocardiographic left ventricular function in sham, myocardial infarct (MI) and MI+spironolactone rats**. (**A**) left ventricular fractional shorting (LV FS), (**B**) LV fractional area change (LV FAC), (**C**) Right ventricular myocardial performance index (RV MPI) and (**D**) Right ventricular tricuspid annulus plane systolic excursion (RV TAPSE). Results are expressed as mean±SEM. *p < 0.0001 vs. Sham; †p < 0.05 vs. Sham.

Echocardiographic LV wall motion abnormality (50 ± 4% vs. 50 ± 5%) and wall motion score index (WMSI) were similar in the MI-untreated and MI+spironolactone groups (table 1). The LV was dilated with increases in LV end-diastolic and end-systolic dimensions and LV end-diastolic and end-systolic areas (p < 0.0001) with no effect of spironolactone treatment (table 1). Compared to sham, LV fractional shortening (FS, 16 ± 2% vs. 53 ± 1%; p < 0.0001, Figure 1A) and fractional area change (FAC, 32 ± 2% vs. 77 ± 2%; p < 0.0001, Figure 1B) were decreased, also without significant effect of spironolactone therapy.

Hemodynamic parameters are presented in table 2. LV end-diastolic pressure (LVEDP) similarly increased in both MI groups but did not reach statistical significance. Indices of LV contractility (LV(+) dP/dt) and relaxation (LV(-) dP/dt) were importantly reduced after MI (p < 0.0001) with no beneficial effect of MR antagonism.

**Table 2:** Hemodynamic parameters

	Sham	MI-untreated	MI+spironolactone
Heart Rate (bpm)	280 ± 12	244 ± 8**	238 ± 6**
MAP (mmHg)	97 ± 7	89 ± 4	84 ± 2†
LVEDP (mmHg)	10 ± 1	16 ± 2	16 ± 2
LV (+) dP/dt (mmHg/s)	7843 ± 491	5121 ± 317*	5156 ± 152*
LV (-) dP/dt (mmHg/s)	-6214 ± 357	-3205 ± 238*	-3164 ± 187*
RV (+) dP/dt (mmHg/s)	1387 ± 92	1688 ± 99	1538 ± 124
RV (-) dP/dt (mmHg/s)	-928 ± 67	-1248 ± 72†	-1133 ± 92

MAP, mean arterial pressure; LV, left ventricular; LVEDP, LV end diastolic pressure; RV, right ventricular.

*p < 0.0001 vs. Sham; **p < 0.01 vs. Sham; †p < 0.05 vs. Sham.

**Figure 2:**
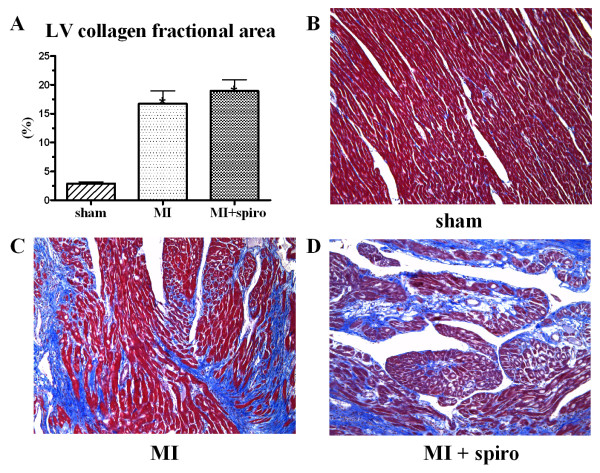
**Effect of spironolactone of myocardial fibrosis in sham, myocardial infarction (MI) and MI+spironolactone rats**. Quantitative analysis of LV collagen fractional area (**A**) and histological LV section with Masson's trichrome staining where blue represent collagen in sham (**B**), MI (**C**) and MI+spironolactone (**D**) groups. Results are expressed as mean±SEM. *p < 0.0001 vs. Sham.

Pathologic LV scar evaluations were performed. Scar weight, scar weight/BW ratio, scar surface and scar weight/scar surface ratio were similar in the MI-untreated and MI-spironolactone groups (table 3). Finally, LV histological study revealed that LV collagen fractional area was highly and similarly increased in MI-untreated and MI+spironolactone groups (17 ± 2%, 19 ± 2%; p < 0.0001) compared to the sham group (2.9 ± 0.3%, Figure 2).

**Table 3:** Effect of spironolactone on BW, heart morphometric and respiratory function parameters

	Sham	MI	MI+spironolactone
Body Weight (g)	431 ± 9	417 ± 8	392 ± 6**‡
Scar Weight (g)	N/A	0.14 ± 0.02*	0.12 ± 0.02**
Scar Weight/Body Weight (%)	N/A	0.033 ± 0.005†	0.04 ± 0.01†
Scar Surface (mm^2^)	N/A	115 ± 9*	111 ± 8*
Scar Weight/Scar Surface (g/mm^2^)	N/A	0.111 ± 0.008*	0.097 ± 0.008*
Respiratory elastance (cmH_2_O/ml)	0.78 ± 0.03	1.2 ± 0.1†	1.2 ± 0.1†
A Parameter, Salazar-Knowles	11.0 ± 0.2	9.6 ± 0.3†	9.3 ± 0.4**
B Parameter, Salazar-Knowles	17.9 ± 0.7	13.8 ± 0.8**	13.5 ± 0.8**
K Parameter, Salazar-Knowles	0.173 ± 0.005	0.148 ± 0.006†	0.145 ± 0.006†

A, estimate of the inspiratory capacity; B, estimate of total lung capacity (p = 0); K, curvature parameter of P-V loop.

*p < 0.0001 vs. Sham; **p < 0.01 vs. Sham; †p < 0.05 vs. Sham; ‡p < 0.05 vs. MI.

### Effects of spironolactone on PH and the development of RV dysfunction and hypertrophy

**Figure 3:**
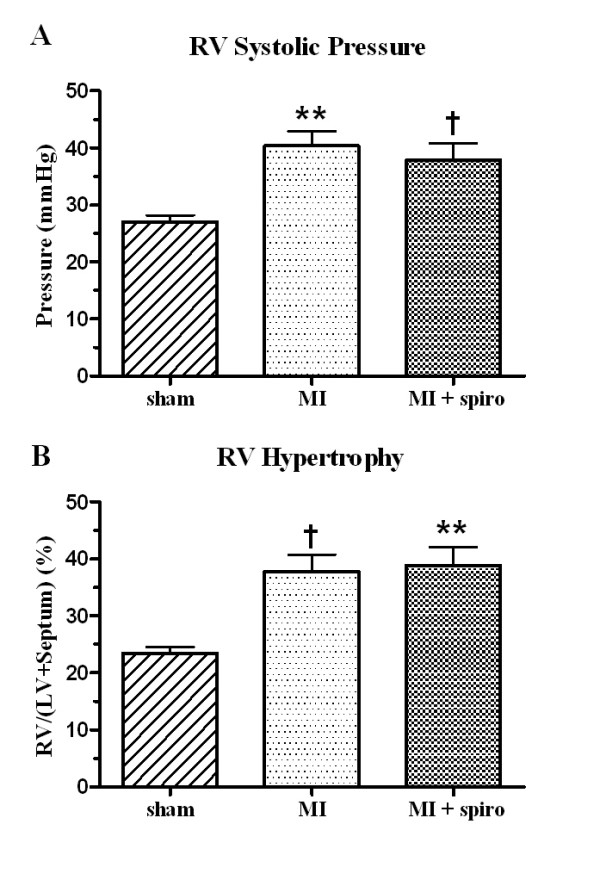
**Pulmonary hypertension and RV hypertrophy in sham, myocardial infarct (MI) and MI+spironolactone**. (**A**) right ventricular systolic pressure (RVSP) and (**B**) right ventricular hypertrophy (RVH). Results are expressed as mean±SEM. **p < 0.01 vs. Sham; †p < 0.05 vs. Sham.

MI-untreated rats developed moderate PH evidenced by a significant increase in RV systolic pressure (40 ± 3 mmHg) compared to sham (27 ± 1 mmHg, p < 0.01). Spironolactone therapy did not modify this increase in RV systolic pressure that remained similarly elevated at 38 ± 3 mmHg (Figure 3A). This elevation of pulmonary pressure in CHF animals was associated with RV hypertrophy revealed by a significant rise of RV/(LV+Septum) weights ratio from 23 ± 1% in sham rats to 38 ± 3% (p < 0.05) in the MI-untreated group. Spironolactone also did not reduce RV hypertrophy (39 ± 3%; p < 0.01, Figure 3B).

Parameters from the echocardiographic examinations of the RV are presented in table 1. There was a non-significant increase of tricuspid valve closing to opening time (TVc-o) in the CHF groups compared to the control group. Similar findings were found for RV myocardial performance index (MPI, Figure 1C). The tricuspid annulus plane systolic excursion (TAPSE), estimating RV systolic function, was significantly and similarly diminished in the CHF groups (Figure 1D).

As previously demonstrated in this model, RV hemodynamic measurements revealed an increase of indices of RV contractility (RV(+) dP/dt, p = ns) and relaxation (RV(-) dP/dt, p < 0.05) in the MI-untreated group, with no effect of spironolactone (table 2).

### Effects of spironolactone on lung structural remodelling and respiratory function

**Figure 4:**
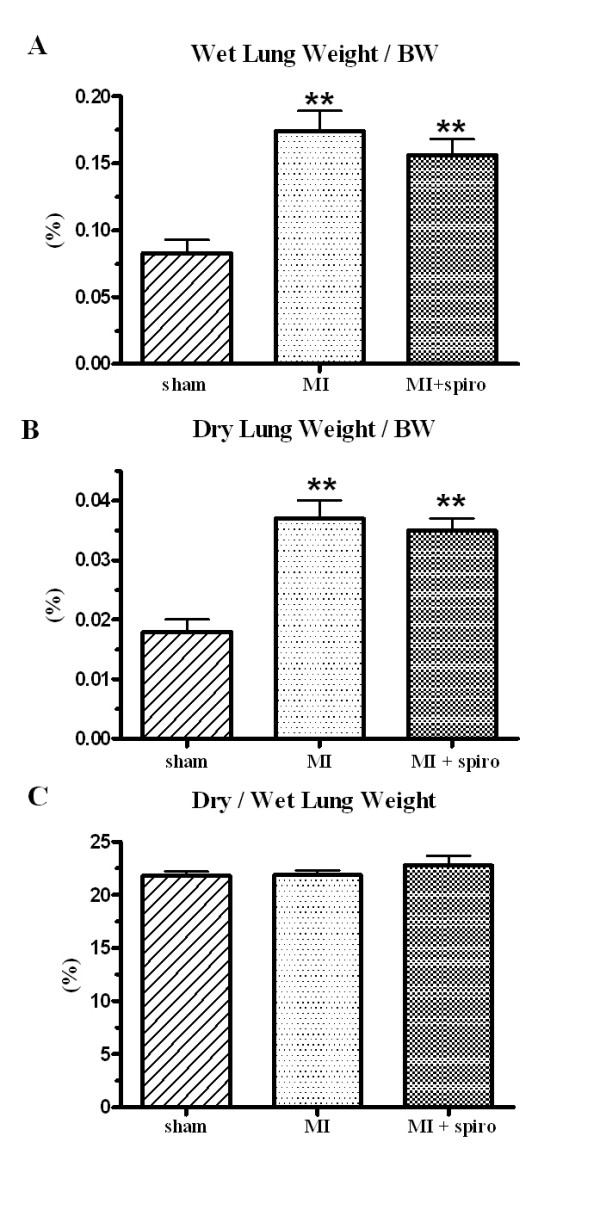
**Lung remodelling in sham, myocardial infarct (MI) and MI+spironolactone rats**. (**A**) wet lung weight/body weight (BW), (**B**) dry lung weight/body weight (BW) and (**C**) dry/wet lung weights. Results are expressed as mean±SEM. **p < 0.01 vs. Sham.

**Figure 5:**
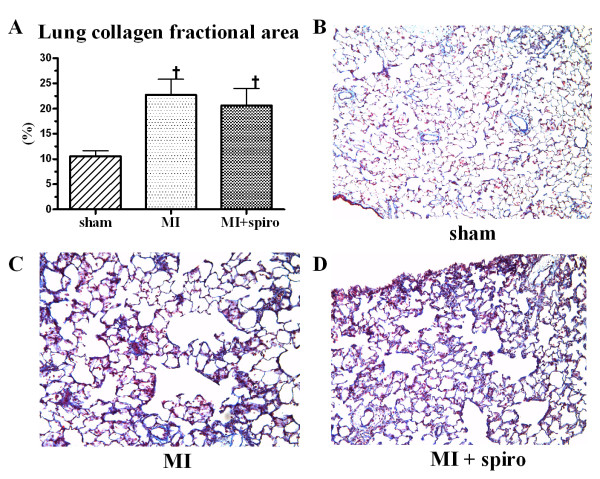
**Lung fibrosis in sham, myocardial infarction (MI) and MI+spironolactone rats**. quantitative analysis of lung collagen fractional area (**A**) and histological lung section with Masson's trichrome staining where blue represent collagen in sham (**B**), MI (**C**) and MI+spironolactone (**D**) groups. Results are expressed as mean±SEM. †p < 0.05 vs. Sham.

CHF almost doubled the ratio of the wet lung weight/BW from 0.08 ± 0.01% in sham rats to 0.17 ± 0.02% in the MI-untreated group (Figure 4A). This increase was the result of important lung structural remodelling since the ratio of the dry lung weight/BW also doubled from 0.018 ± 0.002% in sham rats to 0.037 ± 0.003% in MI-untreated group (Figure 4B). Consequently, there was no significant change in dry/wet lung weight ratio (around 22% for the 3 groups) confirming that no pulmonary oedema was present at this stage of CHF (Figure 4C). Once again, spironolactone therapy did not modify lung structural remodelling. Further evidence of lung remodelling was found at histological studies (Figure 5). Collagen fractional area doubled in the MI-untreated and MI+spironolactone animals (23 ± 3% and 21 ± 3%, respectively) compared to sham (11 ± 1%, p < 0.05).

**Figure 6:**
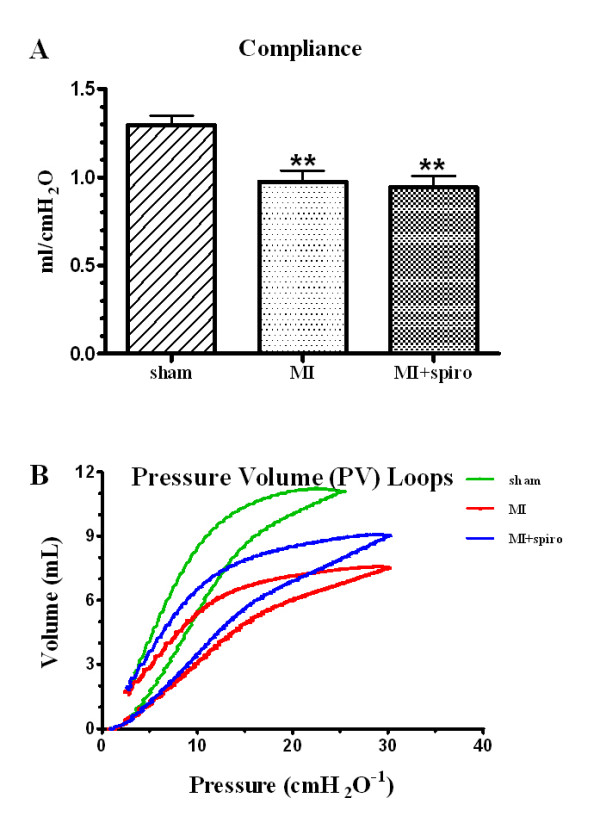
**Respiratory function testing in sham, myocardial infarct (MI) and MI+spironolactone rats**. (**A**) lung compliance and (**B**) respiratory pressure-volume loops. Results are expressed as mean±SEM. **p < 0.01 vs. Sham.

Respiratory function studies revealed that CHF caused a restrictive lung syndrome (Figure 6). There was a decrease (p < 0.01) in respiratory compliance, from 1.30 ± 0.05 ml/cmH_2_0 in sham to 0.98 ± 0.06 ml/cmH_2_0 in the MI group (Figure 6A). However, MR antagonism could not restore lung compliance (0.94 ± 0.06 ml/cmH_2_0). Unsurprisingly, CHF significantly increased respiratory elastance (the inverse of compliance, p < 0.05 vs. sham), which was not reversed by spironolactone (table 3). The respiratory pressure-volume loop depicts this restrictive syndrome with a downward and rightward shift of the curve in CHF (Figure 6B), indicative of lower respiratory volumes at higher inflation pressures. The fitted Salazar-Knowles parameters (A, B and K) were consequently reduced compared to the sham group (table 3). Spironolactone therapy did not reverse the adverse effects of CHF on respiratory function.

**Figure 7:**
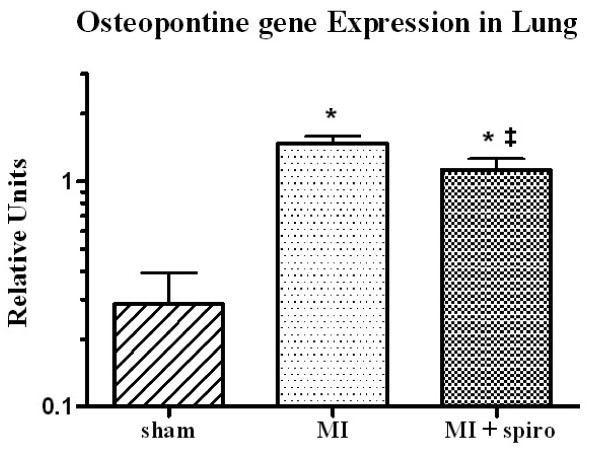
**Effect of spironolactone on lung tissue Osteopontine (OPN) expression in sham, myocardial infarction (MI) and MI+spironolactone rats assessed by T-qPCR**. Results are expressed as mean±SEM. *p < 0.0001 vs. Sham and ‡p < 0.05 vs. MI.

**Figure 8:**
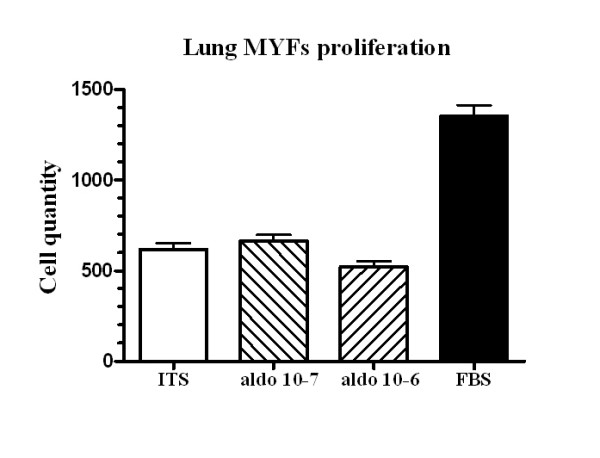
**Lung myofibroblasts (MYFs) proliferation with insulin transferrine selenium (ITS) negative control, aldosterone 10^-7 ^M (**A**), aldosterone 10^-6 ^M (**B**) and fetal bovine serum (FBS) positive control**. Results are expressed as mean±SEM. §p < 0.0001 vs. aldosterone and #p < 0.0001 vs. ITS.

Finally, RT-PCR analysis revealed that CHF induced a 5-fold augmentation of OPN gene expression in the lung (1.5 ± 0.1 relative units, Figure 7) compared to control group (0.3 ± 0.1 relative units, p < 0.0001). MR antagonism succeeded to significantly reduce OPN gene expression in the lung (1.1 ± 0.1 relative units; p < 0.05 vs. MI). Isolated lung MYFs in cell culture revealed that aldosterone treatments for 48 h did not increase lung MYFs proliferation rate (Figure 8).

## Discussion

We evaluated the effects of MR antagonism on pulmonary hypertension, lung structural remodelling and RV function in CHF. After moderate to large MI, rats developed CHF with PH, RV dysfunction, RV hypertrophy and important lung structural remodelling characterized by alveolar wall collagen deposition causing a restrictive respiratory syndrome. Contrary to our hypothesis however, spironolactone therapy had no significant impact on these pathologic modifications. Mechanistically, lung tissue gene expression of the pro-fibrotic mediator OPN was increased in CHF, and reduced in spironolactone-treated animals, but isolated lung MYFs failed to proliferate after aldosterone stimulation. Taken together, these data indicate that aldosterone does not play a central role in promoting lung MYFs proliferation and in contributing to lung structural remodelling and RV dysfunction associated to CHF.

Major clinical trials (RALES, EPHESUS and EMPHASIS-HF) have convincingly demonstrated that the aldosterone antagonists eplerenone and spironolactone reduce mortality and morbidity when added to optimal medical therapy [9-11]. Antagonism of MR is useful, effective and recommended in moderate to severe CHF according to the ACC and AHA guidelines [12,13]. With this study, we had the opportunity to find one possible and as yet unexplored mechanism of the deleterious actions of aldosterone in CHF: its potential effect on lung remodelling and RV function.

Some specific points must be considered when interpreting this negative study by comparison to previous pre-clinical trials evaluating MR antagonism in this CHF model. We were very careful in the timing of therapy and the severity of CHF studied. First, we used a treatment strategy rather than a preventive approach since we started treatment 2 weeks after MI, at a time when CHF is well established and when PH and lung remodelling is already present. Second, we prospectively selected medium to large MIs based on 24 h troponin T measurements. Using this previously validated approach, we randomized rats with established CHF and already evident lung remodelling. Third, we specifically evaluated lung and RV pathology and functions as end-points.

Other investigators previously evaluated MR antagonists after MI in rats. Two important distinguishing points should be noted: first, none of these studies selected animals with more severe CHF, PH and lung remodelling and; second, none specifically studied lung pathology and function. Lal et al. administered spironolactone (80 mg/kg/day) starting 1 to 3 days only after MI for 6 weeks [22]. This "prevention" study found that early administration could restore heart function by improving LV hemodynamics and preventing LV and RV dilatation. In another prevention study, Takeda et al. used 100 mg/kg/day of spironolactone started immediately after MI for 2 weeks and also found that treatment significantly improved LV function and reduced interstitial myocardial fibrosis [23]. Another study using a lower dose of spironolactone (20 mg/kg/day) started immediately after MI for 1 month demonstrated no significant impact on LV function and infarct size [24]. These previous prevention studies therefore suggest that early therapy after MI is beneficial, at least in part, by preventing LV remodelling and improving LV function but that the lower dosage of 20 mg/kg/day is ineffective in rats.

There was only one previous "treatment" study in the rat MI model: Mulder et al. used 80 mg/kg/day of spironolactone started 8 days after MI for a duration 90 days [25]. This is the only study, aside from ours, which left some time for CHF to develop and their results were in agreement with some of our findings. The authors found no effect of therapy on LV hemodynamics and function, although there was a significant effect on LV collagen density measured by Sirius Red staining. A potential explanation for the difference with our study on this aspect might be that the LV collagen deposition can be reduced after 8 days, but not after 14 days and that infarct size may play an important role as large infarct may be less influenced by MR blockade due to a more important activation of other pro-fibrotic mediators. In conclusion, our results do not contradict the pre-clinical evidence suggesting the importance of early MR antagonism in ischemic CHF to prevent LV remodelling and dysfunction. Our results confirm little if any benefit of administration on LV remodelling and function when administered later in the disease process and establish for the first time the lack of effect on established lung remodelling and function and on RV dysfunction.

A negative study always raises the possibility of methodological difficulties that would render the study inconclusive. The dosing of spironolactone was chosen based of previously demonstrated effective dosing regimens. We in fact did find a significant biological effect of spironolactone since lung OPN gene expression was reduced by therapy. OPN is a glycosylated phosphoprotein playing a role in tissue repair and remodelling. OPN is expressed constitutively in healthy lungs and there is prominent OPN expression in lung fibrosis [26]. The expression of OPN has been shown essential for MYFs differentiation [27]. The reduction of OPN expression by spironolactone in our model was evidently insufficient to reverse remodelling suggesting that other pathways would be predominant. Furthermore, we previously demonstrated, using the same model and temporal strategy, that the HMG-CoA reductase inhibitor Atorvastatin could importantly reduce lung remodelling and fibrosis in this model while reducing isolated lung MYF proliferation. Atorvastatin also increased total lung eNOS protein expression compared to CHF suggesting that structural remodelling could be modulated through Rho-kinase and eNOS pathways. On the other hand, the endothelin receptor antagonist Bosentan, used at a dosage effective in models of pulmonary arterial hypertension, also failed to improve lung structural remodelling and PH in our CHF model. Taken together, these results suggest that in advanced stages of CHF with established lung remodelling and fibrosis, blockade of MR receptors provides no significant benefit on respiratory function and PH. Therefore, other pro-fibrotic and proliferation pathways are more important than aldosterone in the maintenance of pathological lung remodelling in CHF.

Our results are not generalizable and must be interpreted in the particular context of the study design. In particular, spironolactone was tested alone to isolate the effect of MR blockade. In human chronic CHF, spironolactone or eplerenone are generally used as add-on therapy on top of angiotensin-converting enzyme inhibitors or angiotensin receptor antagonists, beta-blockers, diuretics and sometimes digitalis. Although we deem this unlikely, MR antagonist as add-on therapy may benefit form synergistic effects and provide some benefit on lung remodelling if tested in that context. Our results are evidently not applicable to patients receiving MR antagonism started early after MI, at a time when myocardial remodelling is occurring and prior to lung remodelling. As discussed above, pre-clinical studies would suggest maximal benefit to these patients. Furthermore, we previously demonstrated that irbesartan, an angiotensin II receptor antagonist, had completely reversed the PH, RVH and lung remodelling consequent to CHF when started immediately after MI [3].

## Conclusions

Chronic moderate to severe CHF in rats causes lung structural remodelling with interstitial fibrosis producing a restrictive respiratory syndrome contributing to RV dysfunction. MR antagonism with spironolactone does not reduce these pathologic modifications. Consequently, aldosterone does not significantly contribute to pulmonary remodelling and RV dysfunction associated with CHF. Other mechanisms of action must be responsible for the beneficial effects of spironolactone in chronic CHF.

## Competing interests

The authors declare that they have no competing interests.

## Authors' contributions

AC contributed to the technical realization of all phases of the project and to data analysis and writing of the first draft of this manuscript. BHJ supervised the histological studies, the lung function studies and the molecular biology experiments. YFS performed the echocardiographic studies. JCT supervised and analysed the echocardiographic studies. JD designed the study, coordinated the research team and contributed to data analysis and final draft of the manuscript. All authors read and approved the final manuscript.

## Pre-publication history

The pre-publication history for this paper can be accessed here:

http://www.biomedcentral.com/1471-2261/11/72/prepub
